# PB.06: Preliminary results in the performance comparison between the size ratio and colour scoring breast ultrasound elastographic techniques

**DOI:** 10.1186/bcr3508

**Published:** 2013-11-08

**Authors:** LCH Leong, THL Moey, LSJ Sim

**Affiliations:** 1 Singapore General Hospital, Singapore

## Introduction

There are a few breast ultrasound elastographic techniques that can help with distinguishing malignant breast lesions from benign ones. Two of the better known ones are the size ratio and colour scoring methods. The aim of this study is to prospectively compare the diagnostic performance of these two elastographic techniques.

## Methods

Female patients referred to the radiology department for image-guided breast biopsy were prospectively evaluated with ultrasound elastography prior to biopsy following informed consent. The two elastographic methods were assessed on each breast lesion separately by different radiologists. A size ratio of ≥1.1 was taken to be malignant and <1.1 was benign. Colour scores of 1 to 3 were taken as benign and colour scores of 4 to 5 were considered malignant. Histological diagnosis was used as the gold standard. The sensitivity and specificity of both techniques were compared using the Fisher's exact test.

## Results

Sixty-five breast lesions in 63 women were evaluated at the interim stage of the study. There were 21 malignant and 44 benign breast lesions. The sensitivity and specificity of the size ratio technique were 100% (21/21) and 81.8% (36/44). The sensitivity and specificity of the colour scoring method were 57.1% (12/21, *P *= 0.001) and 86.4% (38/44, *P *= 0.772).

## Conclusion

The preliminary results indicate that the size ratio elastographic technique is more sensitive and accurate than the colour scoring method. A larger study is ongoing.

